# 

**Published:** 1904-12-15

**Authors:** 


### CONTENTS.

An Ideal Laboratory, *By Charles H. Worboys, D.D.S.* 595

A Modern Operative Equipment, *By E. A. Honey, D.D.S.* 601

The Social Standing of Dentists, *By James Truman, D.D.S.* 605

Rational Treatment of Decomposing Pulps, *By J. P. Buckley, D.D.S.* 609

Adhesion of Vulcanite to Metallic Bases, *By Walter M. Bartlett, D.D.S.* 613

In Sections or Otherwise, *By J. D. Patterson, D.D.S.* 620

Facial Neuralgia, - - *By Dr. Jas. E. Powers.* 622

The New Era in Dentistry, - *By Dr. C. M. Wright.* 625

EDITORIAL, - - - - - - - - 627

ANNOUNCEMENTS, - - - - - - - 629

INDENTS, - - - - - - - - - 631

